# Probiotic and Metabolic Characterization of Vaginal Lactobacilli for a Potential Use in Functional Foods

**DOI:** 10.3390/microorganisms9040833

**Published:** 2021-04-14

**Authors:** Margherita D’Alessandro, Carola Parolin, Danka Bukvicki, Lorenzo Siroli, Beatrice Vitali, Maria De Angelis, Rosalba Lanciotti, Francesca Patrignani

**Affiliations:** 1 Department of Agricultural and Food Sciences, University of Bologna, 47521 Cesena, Italy; lorenzo.siroli2@unibo.it (L.S.); rosalba.lanciotti@unibo.it (R.L.); francesca.patrignani@unibo.it (F.P.); 2 Department of Pharmacy and Biotechnology, University of Bologna, 40127 Bologna, Italy; carola.parolin@unibo.it (C.P.); b.vitali@unibo.it (B.V.); 3 Faculty of Biology, Institute of Botany and Botanical Garden “Jevremovac”, University of Belgrade, 11000 Belgrade, Serbia; dankabukvicki@gmail.com; 4 Interdepartmental Center for Industrial Agri-Food Research, University of Bologna, 47521 Cesena, Italy; 5 Department of Soil, Plant, and Food Sciences, University of Bari Aldo Moro, 70126 Bari, Italy; maria.deangelis@uniba.it

**Keywords:** probiotic, functional characterization, *Lactobacillus crispatus*, *Lactobacillus gasseri*, *Limosilactobacillus vaginalis*, biolog

## Abstract

The main aim of this work was to verify the metabolic and functional aptitude of 15 vaginal strains belonging to *Lactobacillus crispatus*, *Lactobacillus gasseri*, and *Limosilactobacillus vaginalis* (previously *Lactobacillus vaginalis*), already characterized for their technological and antimicrobial properties. In order to evaluate the metabolic profile of these vaginal strains, a phenotype microarray analysis was performed on them. Functional parameters such as hydrophobicity, auto-aggregation, deconjugation of bile salts, adhesion to an intestinal cell line (Caco-2), and a simulated digestion process were evaluated for these strains. A good number of these strains showed hydrophobicity values higher than 70%. Regarding the auto-aggregation assay, the most promising strains were *L. crispatus* BC9 and BC1, *L. gasseri* BC10 and BC14, and *L. vaginalis* BC17. Moreover, *L. crispatus* BC4, BC6, BC7, and BC8 were characterized by strong bile salts hydrolase activity (BHS). In addition, *L. crispatus* BC8 and *L. vaginalis* BC17 were characterized by a medium ability to adhere to Caco-2 cells. Data related to digestion process showed a strong ability of vaginal lactobacilli to withstand this stress. In conclusion, the data collected show the metabolic versatility and several exploitable functional properties of the investigated vaginal lactobacilli.

## 1. Introduction

Traditionally, probiotics, defined as functional microorganisms with a positive impact on human health, have been isolated from the gut. Nevertheless, in recent years, the literature has pointed out the functional role of microorganisms isolated also from other human sources such as the skin, oral cavity, and genital tract, especially for their use as pharmaceutical preparations [1]. In particular, some authors have highlighted the important role of the vaginal microbiome both for the preservation of women’s health and for the creation of a suitable environment for conception and the development of healthy pregnancies [2]. The vaginal microbiome of healthy women consists of a variety of anaerobic and aerobic bacteria with an emphasis on *Lactobacillus*, such as *L. crispatus*, *L. jensenii*, *L. gasseri*, *Limosilactobacillus vaginalis*, and *L. iners* [3,4]. These bacteria act against urogenital pathogens by producing antimicrobial compounds or through competition for adherence to the vaginal epithelium [5,6,7]. In the last decade, the use of bacterial strains characterized by probiotic features in order to prevent vaginal disbyosis or restore normal vaginal microbiome has been proposed [6]. Such beneficial strains can be administered in situ or orally, given their capability to pass from the intestine to the vagina because of spatial proximity of the two apparati [3,8,9,10]. Some strains belonging to the species *L. crispatus*, *L. gasseri,* and *L. vaginalis* were isolated by [7] from the vagina of healthy women and later characterized for their anti-*Candida* [7,11], anti-*Chlamydia trachomatis* [12,13], anti-*Neisseria gonorrhoeae* [14], anti-Group B *Streptococcus* [15], and anti-HIV1 activities [16,17]. In addition, some important technological and safety features were observed on the same collection of lactobacilli (*L. crispatus*, *L. gasseri*, and *L. vaginalis*) [18], pinpointing their significant antimicrobial properties also against foodborne pathogens. Moreover, these strains showed also the aptitude to grow in milk and to produce in this food matrix specific volatile molecule profiles allowing the selection of some strains for their potential future application as functional additional cultures in the dairy sector. In fact, although already available as oral preparations, it would be very challenging to use them in foods as a dietary strategy to prevent human diseases. However, the use of selected microbial strains claimed as probiotics, also in food preparations, cannot avoid their deep functional and metabolic characterization. Therefore, the main aim of this research was to investigate the metabolic and some functional aptitudes of 15 vaginal *Lactobacillus* strains, already characterized for their antimicrobial [7,11,12,14,15,16,17] and technological features of interest for food sector. For this, some properties such as fermentation ability in different substrates, and the ability to maintain high viability in food matrix during refrigerated storage were also tested for their further application as functional cultures in food products. Consequently, in order to dissect their metabolic potential, a Biolog phenotype microarray analysis was applied. Furthermore, the strains were studied for their hydrophobicity, auto-aggregation, the ability to deconjugate bile salts, and the ability to adhere to a human intestinal cell line. Indeed, the ability to survive a simulated digestion process when inoculated in milk and stored at 4 °C was observed.

## 2. Materials and Methods

### 2.1. Strains

For the present study, 15 *Lactobacillus* strains belonging to the collection of FABIT (Department of Pharmacy and Biotechnology, University of Bologna, Italy) were used (Table 1). The strains were isolated from the vagina of pre-menopausal Caucasian women (aged 18–45 years), with no symptoms of vaginal or urinary tract infections, in accordance with the Ethics Committee of the University of Bologna (52/2014/U/Tess). These vaginal strains were also compared with *Lacticaseibacillus rhamnosus* GG ATCC^®^ 53103™, used as a probiotic reference strain. Lactobacilli were cultured in MRS broth (Oxoid Ltd., Basingstoke, UK) with 0.05% L-cysteine and incubated at 37 °C for 24 h in anaerobiosis (GasPak System; Oxoid Ltd., Basingstoke, UK).

### 2.2. Biolog Phenotype Microarray Analysis

Biolog AN plates (Biolog Inc., Hayward, CA, USA) were used to evaluate the catabolic profiles of *L. crispatus* BC3, BC4, BC6, BC7, BC8, *L. gasseri* BC9, BC11, BC13, BC14, and *L. vaginalis* BC16 according to the manufacturers’ instructions. Cells were initially grown in MRS supplemented with 0.05% L-cysteine for 24 h, then Biolog AN plates were inoculated with 150 mL of bacterial suspensions adjusted to 65% transmittance as recommended by the manufacturer. Positive reactions were automatically recorded using a microplate reader with a 590 nm wavelength filter. Negative control measurement was obtained from a well containing water [19].

### 2.3. Hydrophobicity

The hydrophobicity, i.e., the ability to adhere to hydrocarbons, was assessed according to [20] with some modifications. Fresh cultures of the strains (24 h of incubation at 37 °C in MRS broth with 0.05% L-cysteine) in anaerobic conditions (GasPak System; Oxoid Ltd., Basingstoke, UK) were harvested in the stationary phase by centrifugation at 6000 rpm for 10 min. The pellet was resuspended in NaCl 0.9% isotonic solution and was subsequently diluted to the absorbance value of 1 at 560 nm using a spectrophotometer (model 6705, Jenway, Stone, UK). Then, 3 mL of the bacterial suspension was vortexed with 0.6 mL of n-hexadecane (Sigma-Aldrich, Milan, Italy) for 4 min. The two phases were allowed to separate for 1 h at 37 °C. The aqueous phase was removed, and the absorbance (A) at 560 nm was measured. Finally, the hydrophobicity percentage was calculated with the following formula: (A0 − At)/A0 × 100, where A0 represents the absorbance at time 0 and At represents the absorbance at 560 nm after 1 h of incubation at 37 °C.

Hydrophobicity was also evaluated for *L. rhamnosus* GG ATCC^®^ 53103™, a commercial probiotic strain used as reference strain.

### 2.4. Auto-Aggregation Assay

An auto-aggregation assay was performed according to [21], modified by [22]. Fresh cultures of the strains (24 h of incubation at 37 °C in MRS broth with 0.05% L-cysteine) were centrifuged (6000 rpm, 10 min). After removing the supernatant, the pellet was resuspended by NaCl 0.9% isotonic solution to the original volume, using the vortex for 10 s. This assay was determined during 5 h of incubation at room temperature. Every hour, 0.1 mL of the upper suspension was taken and placed in a 0.9 mL NaCl 0.9% isotonic solution, and the absorbance (A) was measured at 600 nm using a spectrophotometer (model 6705, Jenway, Stone, UK). The auto-aggregation was expressed as a percentage according to the formula: 1 − (At/A0) × 100, where At represents the mean of absorbance values at time t = 1, 2, 3, 4, or 5 h, and A0 the absorbance at t = 0.

Additionally, *L. rhamnosus* GG ATCC^®^ 53103™ was used as reference strain.

### 2.5. Bile Salts Deconjugation

The ability of lactobacilli to deconjugate bile salts was investigated by modifying the procedure of [22]. Cultures were screened for bile salt hydrolase (BSH) activity by spotting 10 μL of a culture grown onto BSH agar plates. The plates were prepared with MRS (Oxoid Ltd., Basingstoke, UK) with 0.05% L-cysteine added with 16 g/L of Agar Technical (Oxoid Ltd., Basingstoke, UK), combined with 0.5% (*w*/*v*) sodium salt of taurodeoxycholic acid (Sigma, Milan, Italy) and 0.37 g/L of CaCl_2_ (Sigma, Milan, Italy). Plates were incubated in anaerobic conditions (GasPak System; Oxoid Ltd., Basingstoke, UK) at 37 °C for 48 h; after this time, BSH activity of each strain was evaluated by measuring the diameter of precipitation on screening medium.

### 2.6. Caco-2 Cell Adhesion Tests

In order to describe the ability of *Lactobacillus* strains to adhere to the intestinal epithelium, the Caco-2 cell line was used. This cell line is derived from human colorectal adenocarcinoma and presents the ability to differentiate into cells with many properties typical of enterocytes [23]. Caco-2 cells were routinely grown in DMEM high glucose medium (Sigma-Aldrich, Milan, Italy) with the addition of 2 mM L-glutamine (Sigma-Aldrich, Milan, Italy) and 20% *v*/*v* Bovine Fetal Serum (Sigma-Aldrich, Milan, Italy), in flasks for cell cultures (Corning, Corning, NY, USA), at 37 °C with 5% CO_2_. To obtain differentiated Caco-2 cultures, the cells were inoculated at a density of 10^5^ cells/cm^2^ and kept in culture for 21 days, changing the culture medium every 3–4 days. For adhesion tests, Caco-2 cells were inoculated on sterile glass coverslips in 6-well plates and grown until differentiated. Cells were then incubated with exponentially growing lactobacilli cells by applying a 1:400 ratio, at 37 °C with 5% CO_2_ for 1 h, and washed twice with PBS to remove non-adherent lactobacilli. Samples were then fixed with methanol for 10 min and stained with Giemsa 10% (Sigma-Aldrich, Milan, Italy) for 8 min. Afterwards, samples were washed three times with PBS and then dried in air and observed by an optical microscope (1000× magnification). The adhesion of lactobacilli to Caco-2 cells was evaluated by counting the number of adherent *Lactobacillus* cells to Caco-2 cells, considering at least 200 Caco-2 cells. *L. rhamnosus* GG ATCC^®^ 53103™ was used as the reference strain.

### 2.7. Strain Tolerance to Simulated Digestion Process in Milk

In order to evaluate the resistance of the vaginal strains and *L. rhamnosus* GG ATCC^®^ 53103™, used as reference strain, to the passage through the stomach and duodenum, the method proposed by [24] with certain modifications was performed. Briefly, to test each strain, two identical samples were prepared containing UHT bovine milk with an inoculum of 8–9 log CFU/mL. The first one was used to perform the simulated gastro-duodenal digestion test immediately; the second one was incubated at 4 °C for 7 days and then subjected to the same test. The first sample was mixed with the same volume of a “saliva–gastric” solution. The saliva–gastric solution contained CaCl_2_ (0.22 g/L), NaCl (16.2 g/L), KCl (2.2 g/L), NaHCO3 (1.2 g/L), and 0.3% (*w*/*v*) porcine pepsin (Sigma, Milan, Italy). The sample was quickly brought to pH values of 2.5–3 with HCl 1 M and then was moved into a thermostatic bath for 90 min at 37 °C (WB-MF, Falc Instruments, Treviglio, Italy). After this, 1 mL of sample was taken for the first sampling of the cells’ viability (gastric digestion). In addition, 2 mL of the sample were centrifuged (12,000 rpm, 4 min and 4 °C). After removing the supernatant, the microbial pellet was washed with 2 mL of NaCl 0.9% isotonic solution (12,000 rpm, 4 min and 4 °C). The microbial pellet was resuspended in 2 mL of solution of bile extract porcine (Sigma-Aldrich, Milan, Italy) at a concentration of 1% in PBS, which simulated the hepatic bile. The sample was placed in a thermostatic bath at 37 °C for 10 min in order to simulate the duodenal shock phase of the bile. Then, 100 μL of the sample was taken for the third sampling in order to verify the cells’ viability (duodenal shock). The remaining part of sample was subjected to centrifugation at 12,000 rpm for 4 min at 4 °C. Once the supernatant was removed, the microbial pellet was resuspended in 1.9 mL of NaCl 0.9% isotonic solution and centrifuged under the same conditions. After that, a third solution, representing enteric stress, was added. This solution consists of 0.3% bile and 0.1% pancreatin from porcine pancreas (Sigma-Aldrich, Milan, Italy) dissolved in PBS. The incubation time in the thermostatic bath was 90 min at 37 °C. Then, 100 μL was taken from the sample for the last sampling (intestinal digestion). Samples were plated on MRS agar plates with 0.05% L-cysteine and incubated at 37 °C for 24–48 h in anaerobiosis (GasPak System; Oxoid Ltd., Basingstoke, UK). Cell viability was assessed by the plate count method, and the results were expressed as log CFU/mL.

### 2.8. Statistical Analysis

In order to show the metabolic differences among *L. crispatus* BC3, BC4, BC6, BC7, BC8, *L. gasseri* BC9, BC11, BC13, BC14, and *L. vaginalis* BC16, a heat map analysis was performed using the software found at http://www.heatmapper.ca/, accessed on 7 January 2021 [25]. The average linkage clustering specifying the distance between two clusters was computed as the average distance between objects from the first cluster and objects from the second cluster. All the experimental data are expressed as the mean value of six repetitions. The data were statistically analyzed by Statistica software (version 8.0; StatSoft, Tulsa, OK, USA) and subjected to the analysis of variance (ANOVA), and the test of mean comparison, according to Fisher’s least significant difference (LSD), was applied on all obtained data. The level of significance was *p* < 0.05.

## 3. Results and Discussion

### 3.1. Phenotype Microarray Analysis

**Figure 1:**
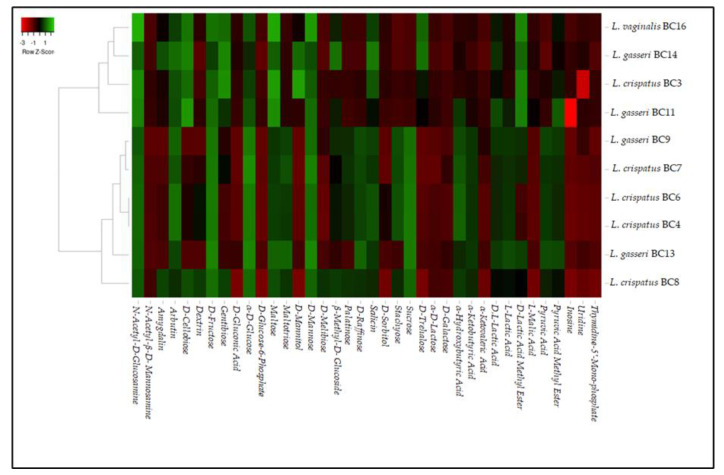
Heat map showing the metabolic differences among *L. crispatus* BC3, BC4, BC6, BC7, BC8, *L. gasseri* BC9, BC11, BC13, BC14, and *L. vaginalis* BC16 with green referring to high catabolic activity and red to low activity.

Biolog phenotype microarray analysis was performed on *L. crispatus* BC3, BC4, BC6, BC7, *L. gasseri* BC9, BC11, BC13, BC14, and *L. vaginalis* BC16 strains in order to evaluate their metabolic profiles in relation to different carbon sources. Raw data were analyzed by a heatmap reported in Figure 1. According to the obtained heatmap, the analyzed strains were grouped into two clusters: one including *L. gasseri* BC11 and BC14 strains, *L. vaginalis* BC16, and *L. crispatus* BC3 and another one including most *L. crispatus* strains and *L. gasseri* BC9 and BC13. In particular, the two clusters were created for the different metabolic behaviors against pyruvic acid, pyruvic acid methyl ester, L-lactic acid, α-ketobutyric acid, α-hydroxybutyric acid, D-trehalose, sucrose, stachyose, D-raffinose, palatinose, and D-mannitol. The data obtained lead to considerations about some features showed by these strains. First of all, all the strains were characterized by a strong catabolic activity against α-D-glucose, D-fructose, maltose, and D-mannose. Furthermore, the majority of the strains were also able to use gentiobiose, maltotriose, D-raffinose, stachyose, and sucrose. This phenotypic approach could be considered a useful starting point to characterize several strains and to individuate their specific metabolic properties to be considered for their inclusion in a food matrix. In fact, the preliminary metabolic data, in addition to highlighting the potential of the strains as starters, co-starters, or adjuncts, can give important information regarding the characteristics that they can impart to the final products. On the other hand, in relation to the metabolic profiles characterizing a specific microbial strain, a proper and tailored use in relation to the characteristic of the food matrix can be exploited. As regards the ability to use n-acetyl-D-glucosamine, shown by all the strains, this quality is of potential interest as this compound is the monomer unit of chitin, the second most abundant carbohydrate after cellulose. N-acetyl-D-glucosamine is also a basic component of hyaluronic acid and keratin sulfate on the cell surface [26]. On the other hand, most of these selected vaginal lactobacilli were also able to catobolize D-cellobiose and α-ketobutyric acid. The use of cellobiose is remarkable considering that interest in new potential prebiotics such as cello-oligosaccharides has increased [27]. In this sense, a study conducted by [28] investigated the effect of cellobiose on growth rates of probiotics such as *Bifidobacterium* spp. According to these reports, cellobiose has a higher prebiotic index than FOS [29]. Thus, the data obtained in this research suggest that some strains could be exploited for the formulation of symbiotic functional foods in which probiotics and prebiotics (deriving from the metabolic activities of added probiotics) are simultaneously present. Other disaccharides such as amygdalin, D-melibiose, and D-trehalose as unique carbon sources were used in a strain-dependent way, with the exception of arbutin that was used by all the tested strains. However, the utilization of these disaccharides implies the presence of various hydrolytic enzymes that enable them to break down the substrate, such as β-glucosidase or β-amylase [30]. Additionally, the ability to catabolize α-ketobutyric acid is significant because this compound is the precursor of 3-hydroxy-4,5-dimethyl-2(5H)-furanone, an aromatic compound responsible for the burnt, sugar, and curry flavor in dairy products [31]. Regarding the growth kinetics on α-D-lactose, D-galactose, and D-trehalose, almost all strains showed low efficiency of use.

### 3.2. Hydrophobicity and Auto-Aggregation

**Figure 2:**
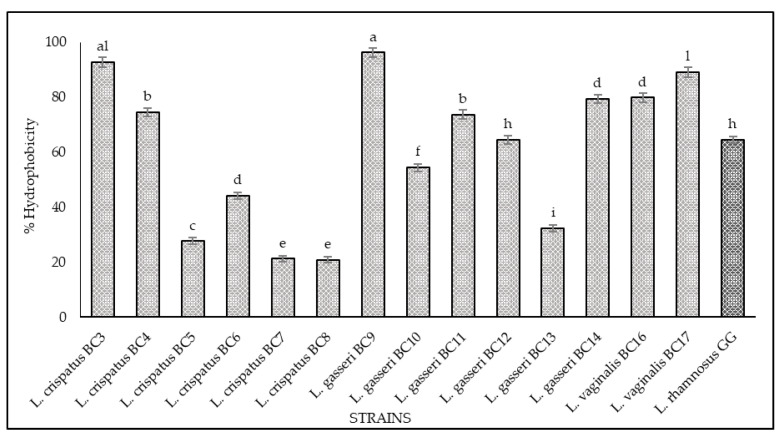
Cell hydrophobicity of *L. crispatus* BC3, BC4, BC5, BC6, BC7, BC8, *L. gasseri* BC9, BC10, BC11, BC12, BC13, BC14, *L. vaginalis* BC16, BC17, and *L. rhamnosus* GG ATCC^®^ 53103™. The hydrophobicity percentage was calculated with the following formula: (A0 − At)/A0 × 100, A0 represents the absorbance at time 0 and At represents the absorbance at 560 nm after 1 h of incubation at 37 °C. Results are shown as average ± SD. Samples with different letters are significantly different (*p* < 0.05).

**Figure 3:**
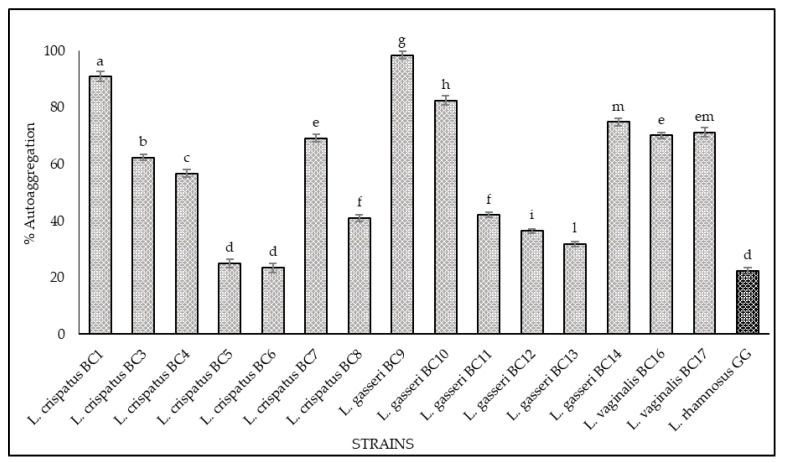
Cell auto-aggregation of *L. crispatus* BC1, BC3, BC4, BC5, BC6, BC7, BC8, *L. gasseri* BC9, BC10, BC11, BC12, BC13, BC14, *L. vaginalis* BC16, BC17, and *L. rhamnosus* GG ATCC^®^ 53103™. The auto-aggregation was expressed as a percentage according to the formula: 1 − (At/A0) × 100, where At represents the average of absorbance values at time t = 1, 2, 3, 4, or 5 h and A0 the absorbance at t = 0. Samples with different letters are significantly different (*p* < 0.05).

The results of hydrophobicity and auto-aggregation assays are reported in Figure 2 and Figure 3, respectively. Cell surface hydrophobicity and autoaggregation were evaluated also for *L. rhamnosus* GG ATCC^®^ 53103™, a recognized probiotic strain used as a reference. As shown, it was observed that the vaginal strains exhibited a different degree of hydrophobicity (Figure 2). The highest values were obtained for *L. gasseri* BC9 (96.23%), *L. crispatus* BC3 (92.8%), *L. vaginalis* BC17 (89.08%), *L. vaginalis* BC16 (79.83%), and *L. gasseri* BC14 (79.32%). The strains *L. crispatus* BC4, *L. gasseri* BC11, and *L. gasseri* BC12 showed hydrophobicity values of 74.57%, 73.70%, and 64.63%, respectively, while the remaining strains expressed levels below 60%. Regarding the auto-aggregation assay, the most promising strains were *L. gasseri* BC9 (98.49%), *L. crispatus* BC1 (90.86%), *L. gasseri* BC10 (82.43%), *L. gasseri* BC14 (74.87%), *L. vaginalis* BC17 (71.74%), *L. vaginalis* BC16 (70.14%), and *L. crispatus* BC7 (69.17%) (Figure 3). *L. crispatus* BC3 and BC4 strains showed a rate of auto-aggregation of 62.4% and 56.72%, respectively, while the remaining strains indicated percentages below 40%. These considerations are even more relevant when considering that the hydrophobicity and auto-aggregation values reported for many of the vaginal strains are better than those recorded for GG ATCC^®^ 53103™ (64% hydrophobicity and 23% autoaggregation), which is a commercial probiotic strain. The data obtained lead to considerations about the functional properties of these strains since the hydrophobic nature of the surface of microorganisms can be related to the attachment of bacteria to host tissues [21,22,32,33,34] conceding to the microorganisms a competitive advantage, important for their permanence in the human gastrointestinal tract [33,34]. In this context the hydrophobicity percentages for the tested strains ranged between 20% and 96%, and among all the strains, only four showed levels of hydrophobicity below 40%. This achievement is even more remarkable with respect to the studies conducted by [35] and [36], which underlined that lactobacilli were much more hydrophilic, showing values of 40% or less; consequently, all the tested strains with hydrophobicity values more than 40% could be considered as hydrophobic. In addition, the high auto-aggregation capacity detected for many of the tested strains is another key factor for the determination of the ability of the probiotic strain to adhere to the gastrointestinal and urogenital tract [35]. Furthermore, during this study, a strong correlation between these two parameters (hydrophobicity and auto-aggregation) was observed. In fact, similar trends for some strains were recorded, especially for *L. gasseri* BC9, which showed the highest values in terms of hydrophobicity (96.23%) and auto-aggregation (98.49%). According to [35,37] these positive results, lead to better adhesion to intestinal cells.

### 3.3. Bile salts Deconjugation

Regarding the ability of vaginal strains to deconjugate bile salts by bile salt hydrolase (BSH) enzyme activity, the assay was performed according to [22]. This feature is very important for the selection of functional strains since it can permit the reduction of bile toxicity by the deconjugation of bile salts into bile acids [38,39]. According to [40], deconjugated bile salts are less soluble and less efficiently reabsorbed from the intestinal lumen than their conjugated compounds. Within this experimental research, the functional attribute of bile salt deconjugation was observed for *L. crispatus* BC4, *L. crispatus* BC6, *L. crispatus* BC7, and *L. crispatus* BC8. Moreover, our results are not in agreement with [41], which reported that BSH activity has not been detected in bacteria isolated from environments from which bile salts are absent. On the other side, a more recent study by [39] identified BSH-positive LAB isolates not associated with the intestinal environment. With regard to the tested strains not able to deconjugate bile salts, the most widely used LAB in food are not gifted with this functional feature, without excluding their important role as probiotics [41].

### 3.4. Adhesion to Caco-2 Cells

**Figure 4:**
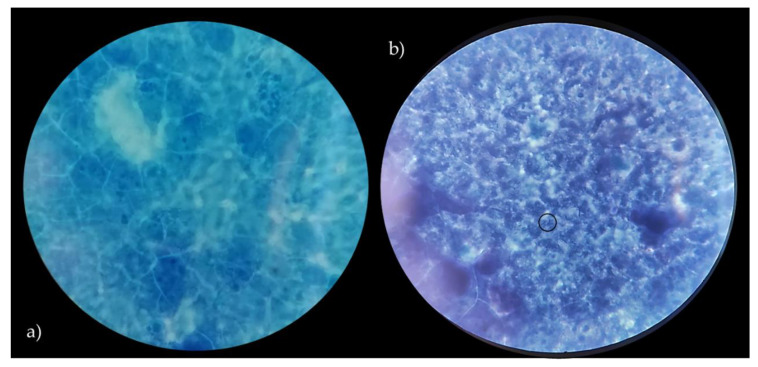
Caco-2 cells with no bacteria (**a**), adhesion of *L. crispatus* BC8 to Caco-2 cells (**b**). Images obtained under the optical microscope at 1000× magnification, in evidence the adherent bacteria.

**Figure 5:**
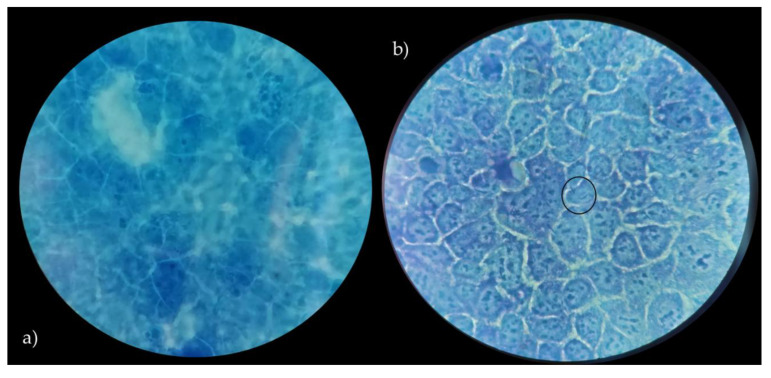
Caco-2 cells with no bacteria (**a**), adhesion of *L. rhamnosus* GG ATCC^®^ 53103™ to Caco-2 cells (**b**). Images obtained under the optical microscope at 1000× magnification, in evidence the adherent bacteria.

In order to assess the ability of *Lactobacillus* strains to adhere to the intestinal epithelium, as a key indicator of adhesion to epithelial cells and to gut mucosal surfaces, Caco-2 cells were used. According to several authors [23,42], Caco-2 cells show significant features of human intestinal cells, including the ability to spontaneously differentiate into a cell monolayer with typical properties of enterocytes with a brush border layer as found in the small intestine. Therefore, human cell line Caco-2, originally derived from a colon adenocarcinoma, has been widely used as a model of the intestinal epithelial barrier [23]. In this context, the data related to the adhesive properties of the strains, expressed as the number of lactobacilli cells on Caco-2 cells, are shown in Table 2. Vaginal lactobacilli were characterized by values of adhesiveness ranging between 0.15 and 5.14 (bacterial cells/Caco-2 cell). Only *L. crispatus* BC8 showed an adhesiveness value of 5.14, while *L. vaginalis* BC17 indicated an adhesion value equal to 2.32. According to the criteria proposed by [42], these two strains can be defined by having a medium ability to adhere to the Caco-2 epithelial monolayer, while the remaining strains can be considered strains with low ability. On the other hand, the two strains with medium aptitude were also characterized by high auto-aggregation and hydrophobicity properties. Moreover, *L. rhamnosus* GG ATCC^®^ 53103™, used as reference strain, showed a higher value of adhesion (8.90) but not significant different from *L. crispatus* BC8. In detail, images of *L. crispatus* BC8 and *L. rhamnosus* GG ATCC^®^ 53103™ to epithelial cells, obtained by optical microscope are shown in Figure 4 and Figure 5. In this context, [43] also highlighted for *L. rhamnosus* GG ATCC^®^ 53103™ a similar trend of adhesiveness to Caco-2 cells. Although the studied strains, with the exception of *L. crispatus* BC8 and *L. vaginalis* BC17, showed an overall low ability to adhere to Caco-2 cells, it is necessary to consider that the gut environment is characterized by a high level of complexity, which includes the presence of factors such as mucus that could furtherly promote the adhesion mechanism in vivo. On the other hand, these vaginal strains were already evaluated for their ability to adhere to HeLa cells, a cell line that originated from a human carcinoma of the cervix [7], demonstrating that this functional property is strain specific rather than species specific.

### 3.5. Strain Tolerance to Simulated Digestion Process in Milk

**Figure 6:**
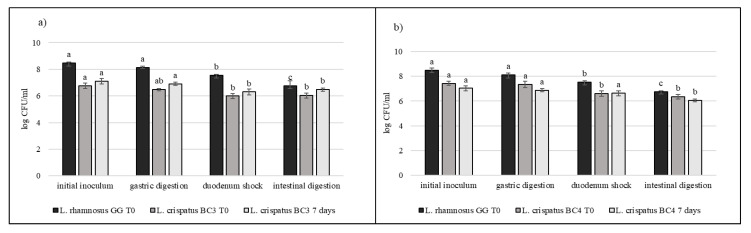
Cell loads of *L. crispatus* BC3 (**a**) and *L. crispatus* BC4 (**b**) after the simulated stomach–duodenum passage, immediately after the inoculation in milk (T_0_) and after 7 days of refrigerated storage, also compared with *L. rhamnosus* GG ATCC^®^ 53103™. Results are shown as average ± SD. Samples with different letters are significant different (*p* < 0.05).

**Figure 7:**
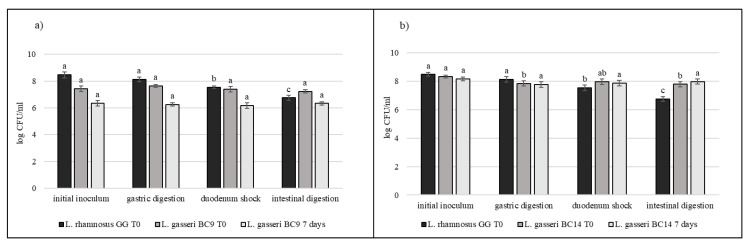
Cell loads of *L. gasseri* BC9 (**a**) and *L. gasseri* BC14 (**b**) after the simulated stomach–duodenum passage, immediately after the inoculation in milk (T_0_) and after 7 days of refrigerated storage, also compared with *L. rhamnosus* GG ATCC^®^ 53103™. Results are shown as average ± SD. Samples with different letters are significant different (*p* < 0.05).

**Figure 8:**
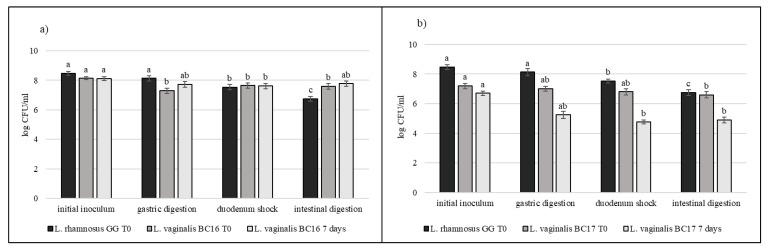
Cell loads of *L. vaginalis* BC16 (**a**) and *L. vaginalis* BC17 (**b**) after the simulated stomach–duodenum passage, immediately after the inoculation in milk (T_0_) and after 7 days of refrigerated storage, also compared with *L. rhamnosus* GG ATCC^®^ 53103™. Results are shown as average ± SD. Samples with different letters are significant different (*p* < 0.05).

Strains selected on the basis of previous results (*L. crispatus* BC3, *L. crispatus* BC4, *L. gasseri* BC9, *L. gasseri* BC14, *L. vaginalis* BC16, and *L. vaginalis* BC17) were studied during the simulated digestion in milk in comparison with *L. rhamnosus* GG ATCC^®^ 53103™, considered a reference strain. Strains were inoculated in milk (8–9 log CFU/mL) and subjected to gastric digestion and duodenal and intestinal shock. The ability of these vaginal lactobacilli to resist this type of stress after inoculation in milk and after 7 days of storage at 4 °C was also assessed (Figure 6, Figure 7 and Figure 8). In general, *L. crispatus* BC3, *L. crispatus* BC4, *L. gasseri* BC14, and *L. vaginalis* BC16 showed a significant maintenance of cell viability after the application of simulated stress and during refrigerated storage. In particular, *L. vaginalis* BC16 showed, following exposure to acid stress (simulating human stomach) immediately after the inoculum, a decrease from 8 log CFU/mL to 7 log CFU/mL, highlighting how gastric stress affects the decrease in cell viability compared to duodenal/intestinal stress. Similar results using a different strain were shown by [44] using the Simulator of the Human Intestinal Microbial Ecosystem (SHIME). On the contrary, *L. rhamnosus* GG ATCC^®^ 53103™, although remaining at high level of cell loads, was more significantly affected by the duodenum and intestinal steps where bile salts are present. As shown in Figure 7, although *L. gasseri* BC9 showed a significant maintenance of cell viability during all digestion steps at time 0, after one week of refrigerated storage, a reduction in cell load from 7 log CFU/mL to 6 log CFU/mL was observed, pointing out the greater sensitivity of this strain to the storage conditions adopted rather than to gastric shock. On the other hand, *L. vaginalis* BC17 showed a great resistance during the simulation of the digestion process at time 0, while after 7 days at +4 °C a significant reduction in cell loads (from 7 log CFU/mL to 5 log CFU/mL) was highlighted following simulated gastric stress, without further loss in terms of viability. In general, the decrease in viability following the applied gastric stress was contained in each case [11]. However, the application of acid shock has certainly induced better resistance in vaginal lactobacilli to the following stress conditions [34,45]. In addition, the milk matrix in which the vaginal strains were carried in certainly had a protective effect against the loss of cell viability, offsetting the negative effects of refrigerated storage. The study conducted by [46] showed that *L. crispatus* BC4 inoculated as adjunctive culture in the Squacquerone cheese (stored at 4 °C for 18 days) demonstrated a good rate of survival during the refrigerated storage and after the simulation of digestion process performed in vitro. Although only in vivo tests can confirm the effective functionality of the food, in terms of prevention and/or treatment of vaginal dysbiosis, these vaginal strains were also evaluated for their ability to adhere to HeLa cells, a cell line that originated from a human carcinoma of the cervix [7]. Regarding this, our data set up initial results for the first functional and technological characterization of selected vaginal strains and conditions for their use in functional foods for preserving or restoring women’s well-being.

## 4. Conclusions

The results obtained in this work highlight the functional potential of *L. crispatus*, *L. gasseri,* and *L. vaginalis* for their further application in food. According to their metabolic profiles, they could be exploited for the formulation of symbiotic foods. The lack of ability to promptly use carbon source as lactose or galactose make them good candidates as adjuncts probiotic cultures in dairy products. The data obtained underlined high values of hydrophobicity and auto-aggregation (over 70%) for most of the vaginal lactobacilli considered in this study. Even compared to *L. rhamnosus* GG ATCC^®^ 53103™, a commercial probiotic strain used as reference, all vaginal strains showed higher values of hydrophobicity and auto-aggregation. In this context, the connection between these two parameters is positively recognized; in fact, the lactobacilli gifted with a hydrophobic cell surface and strong auto-aggregation ability could have a greater chance for adhesion to human cells. In addition, *L. crispatus* BC4, BC6, BC7, and BC8 showed significantly strong BHS enzyme activity. This feature is related to the microbial ability to modulate lipid metabolism and allow the improvement of the survival of these bacteria under the stringent conditions typical of the intestinal tract. However, most of the vaginal lactobacilli herein considered were characterized by low adhesion to a model of intestinal epithelial cells, except for the *L. crispatus* BC8 and *L. vaginalis* BC17 strains, which were characterized by a medium value of adhesion, also compared to *L. rhamnosus* GG ATCC^®^ 53103™ which showed a higher level of adhesion but not significantly different from that of *L. crispatus* BC8. Regarding the resistance of vaginal strains during the simulated digestion process and tested immediately after inoculation in milk and after 7 days of storage at +4 °C, the decrease in viability was limited for all the strains, showing therefore a good resistance even after exposure to gastric acidity (pH 3).

In conclusion, the attained data represent an important contribution to better understanding the proper use of the studied strains in food formulations in order to further use food as dietary strategy to enhance human well-being. In particular, these strains, isolated from healthy women’s vaginas could be thought to be used as probiotics in “gender foods” and particularly to increase women’s wellbeing.

## Figures and Tables

**Table 1:** Vaginal lactobacilli used in the present study.

Strains	Species	Isolation Source	Collection
BC1	*Lactobacillus crispatus*	Female genital tract [13]	FABIT
BC3	*Lactobacillus crispatus*	Female genital tract [13]	FABIT
BC4	*Lactobacillus crispatus*	Female genital tract [13]	FABIT
BC5	*Lactobacillus crispatus*	Female genital tract [13]	FABIT
BC6	*Lactobacillus crispatus*	Female genital tract [13]	FABIT
BC7	*Lactobacillus crispatus*	Female genital tract [13]	FABIT
BC8	*Lactobacillus crispatus*	Female genital tract [13]	FABIT
BC9	*Lactobacillus gasseri*	Female genital tract [13]	FABIT
BC10	*Lactobacillus gasseri*	Female genital tract [13]	FABIT
BC11	*Lactobacillus gasseri*	Female genital tract [13]	FABIT
BC12	*Lactobacillus gasseri*	Female genital tract [13]	FABIT
BC13	*Lactobacillus gasseri*	Female genital tract [13]	FABIT
BC14	*Lactobacillus gasseri*	Female genital tract [13]	FABIT
BC16	*Limosilactobacillus vaginalis*	Female genital tract [13]	FABIT
BC17	*Limosilactobacillus vaginalis*	Female genital tract [13]	FABIT

**Table 2:** Adhesion capacity of the vaginal strains and *L. rhamnosus* GG ATCC^®^ 53103™ on Caco-2 cells. Data are expressed as lactobacilli cells/Caco-2 cell and shown as average ± SD. Samples with different letters are significant different (*p* < 0.05).

Strains	Adhesion (Lactobacilli Cells/Caco-2 Cell)
*Lactobacillus crispatus* BC1	0.63 ^a^ (±0.21)
*Lactobacillus crispatus* BC3	0.45 ^ad^ (±0.19)
*Lactobacillus crispatus* BC4	0.24 ^b^ (±0.09)
*Lactobacillus crispatus* BC5	0.43 ^ad^ (±0.17)
*Lactobacillus crispatus* BC6	0.74 ^a^ (±0.21)
*Lactobacillus crispatus* BC8	5.14 ^c^ (±2.29)
*Lactobacillus gasseri* BC9	0.26 ^d^ (±0.10)
*Lactobacillus gasseri* BC11	0.27 ^d^ (±0.11)
*Lactobacillus gasseri* BC12	0.75 ^a^ (±0.23)
*Lactobacillus gasseri* BC14	0.15 ^e^ (±0.06)
*Limosilactobacillus vaginalis* BC16	0.34 ^ad^ (±0.16)
*Limosilactobacillus vaginalis* BC17	2.32 ^f^ (±1.17)
*Lacticaseibacillus rhamnosus* GG	8.90 ^g^ (±1.24)

## Data Availability

The data presented in this study are available in the article.
